# Computational Indicator Approach for Assessment of Nanotoxicity of Two-Dimensional Nanomaterials

**DOI:** 10.3390/nano12040650

**Published:** 2022-02-15

**Authors:** Alexey A. Tsukanov, Boris Turk, Olga Vasiljeva, Sergey G. Psakhie

**Affiliations:** 1 Center for Computational and Data-Intensive Science and Engineering (CDISE), Skolkovo Institute of Science and Technology (Skoltech), 121205 Moscow, Russia; 2 Institute of Strength Physics and Materials Science of SB RAS, 634055 Tomsk, Russia; 3 Department of Biochemistry and Molecular and Structural Biology, Jozef Stefan Institute, SI-1000 Ljubljana, Slovenia; boris.turk@ijs.si

**Keywords:** two-dimensional nanomaterials, cytotoxicity prediction, membrane disruption, lipid extraction, in silico models

## Abstract

The increasing growth in the development of various novel nanomaterials and their biomedical applications has drawn increasing attention to their biological safety and potential health impact. The most commonly used methods for nanomaterial toxicity assessment are based on laboratory experiments. In recent years, with the aid of computer modeling and data science, several in silico methods for the cytotoxicity prediction of nanomaterials have been developed. An affordable, cost-effective numerical modeling approach thus can reduce the need for in vitro and in vivo testing and predict the properties of designed or developed nanomaterials. We propose here a new in silico method for rapid cytotoxicity assessment of two-dimensional nanomaterials of arbitrary chemical composition by using free energy analysis and molecular dynamics simulations, which can be expressed by a computational indicator of nanotoxicity (CIN_2D_). We applied this approach to five well-known two-dimensional nanomaterials promising for biomedical applications: graphene, graphene oxide, layered double hydroxide, aloohene, and hexagonal boron nitride nanosheets. The results corroborate the available laboratory biosafety data for these nanomaterials, supporting the applicability of the developed method for predictive nanotoxicity assessment of two-dimensional nanomaterials.

## 1. Introduction

In the last two decades, nanomaterials have actively advanced into various applications in different industries. The amounts of produced nanomaterials and their variety are rapidly growing. The use of multifunctional nanoparticles in medical diagnostics and therapy has led to new developments in medicine, such as nanomedicine and theranostics [1,2]. Nanoparticles and nanomaterials are used as drug delivery carriers, contrast agents [3,4,5], and potentiating or independent therapeutic agents [6,7,8]. The primary requirement for nanoparticles used for medical applications as a carrier, bioactive agent, or as a part of the nanohybrid theranostic system is the low toxicity of nanomaterials. Moreover, special attention should be paid to nanomaterials that have been designed to induce cytotoxicity toward particular cells or tissues, for example, tumor cells, while administered systemically [9,10]. Thus, the design and development of new nanomaterials for medical applications require reliable predictive methods to assess their safety.

The range of laboratory test methods for determining the cytotoxicity of novel nanomaterials is rapidly expanding. In vitro cytotoxicity tests use various methods ranging from the simple visual inspection of cell or nuclear morphology by bright field microscopy [11] to the most common cell viability methods. The latter is subdivided into methods for measuring plasma membrane integrity (neutral red and trypan blue assays [12,13], live/dead viability tests with calcein acetoxymethyl and ethidium homodimer [14], lactate dehydrogenase release monitoring [15]), and mitochondrial activity (MTT viability assay [16,17], MTS and WST assays [18,19]). Although in vitro cytotoxicity assays measure the effect of nanoparticles on individual cells, the effect of the same nanoparticles in the complex biological environment of living organisms can be validated only by in vivo studies. The latter approach can enable the evaluation of nanoparticle biodistribution, pharmacokinetics, and accumulation in a living organism and in specific tissues [20]. However, the testing of nanomaterial toxicity in vivo is expensive and requires a large number of animals that are often euthanized after their use. To minimize unnecessary testing in animals, the principles of the 3Rs (Replacement, Reduction, and Refinement) [21] have been introduced and actively incorporated into legislations, guidelines, and practice of animal experiments and research. Nevertheless, despite being officially adopted in multiple legislations and guidelines, the 3Rs principles have not been fully incorporated into everyday toxicity testing in animals. One of the major reasons for the limited implementation of the 3Rs principles in biomedical research is the delay in the development of reliable, and predictive in vitro and, particularly, in silico approaches. In addition, the unfortunate aspect of in vivo pharmacology research is that many animal studies are conducted for testing very early stage substances or screening of multiple compounds, a major part of which would not advance to clinical applications or public consumption and use. Thus, although certain animal studies are required for the final evaluation of medical product safety before its first administration to human, the testing and screening of early stage development compounds could be performed much faster and at a lower cost using advanced in silico models.

Due to the rapid development of a wide variety of chemical compositions and morphological forms of nanomaterials and nanostructures, in silico methods and models have gained special importance for the design of new bioactive nanoagents and prediction of their toxic properties [22,23,24]. The applied approaches include the quantitative structure–activity relationship (QSAR); quantitative nanostructure–activity relationship (QNAR); quantitative structure–property relationship (QSPR); quantitative structure–toxicity relationship (QSTR). QNAR, QSPR, and QSTR methods (hereinafter, group I), which use established relationships between the molecular structure of the nanoagent and its properties or mode of bio-action [25,26,27]. These approaches proved efficient for the development of new drugs from components (e.g., chemical functional groups) with a known effect. The processing and analysis of statistical data and the construction of descriptors–property relationships (dependencies) in group I approaches are conducted using modern computational and data science technologies, including the powerful methods as machine learning and artificial intelligence (for example, ref. [28]).

Molecular docking and metadynamics simulations [29,30] can be assigned to a separate group of in silico methods (group II). This approach relies on direct models and either quantum mechanical (ab initio) or force field-based calculations to define potential binding sites between the nanoagent and biotarget, thus evaluating the free energy of their binding. In drug development, docking simulations of various functional ligands into a biotarget are used to investigate the mechanism of action at the molecular level by showing binding sites and effects on the structure, properties, and functions of the biotarget. Next, the most favorable nanoagent structure and composition can be chosen to provide the best binding with the biotarget and, for example, to inhibit or enhance biotarget activity. In nanoagent toxicity assessment, molecular docking of active nanoagent groups with various peptides, proteins, DNA and RNA fragments, biological ions, etc. reveals the binding mechanism and determines the probable biotarget.

In the third group of in silico methods (group III), classical methods of direct molecular dynamics simulation at the atomic [31,32] and coarse-grained [33,34,35] levels can be included. They model the impact of nanoobjects on the cell or bacterial membrane, as well as integrated membrane proteins, to predict toxicity [35,36,37,38]. Direct modeling of cell membranes, in particular, with embedded proteins, requires significant computational resources due to a large number of involved atoms/particles, which limits its use. This approach presents the most common direct in silico experiment but does not allow a fast selection of nanoagent parameters (e.g., for an iterative screening) because of the considerable time and required resources.

In this study, we describe a novel method for the assessment of nanomaterial interactions with the cell membrane, including cytotoxic activity, by combining the advantages of the aforementioned three in silico approaches applied to two-dimensional (2D) nanomaterials. Based on thermodynamic grounds, our study aims to identify a critical factor associated with toxicity, a nanotoxicity indicator, similarly to the descriptors in group I, that can be rapidly assessed by the direct modeling of nanomaterials at the molecular level, as in group III, and combined with the free energy analysis of functional units interaction, as in group II.

One of the mechanisms of the cytotoxic action of nanomaterials (or nanoparticles) is the disruption of the cell membrane and/or the membranes of intracellular organelles [39,40]. Nanomaterials that do not cause membrane rupture can either be non-toxic or exhibit toxicity by a mechanism not directly related to cell membrane destruction [41]. In this work, we focus on predicting nanomaterial toxicity associated only with cell membrane rupture.

A typical plasma membrane is composed of a double lipid layer containing amphiphilic lipid molecules as the principal structural components. The lipid molecules are oriented in such that both membrane surfaces are hydrophilic and the interior inaccessible to solvent or water is hydrophobic [42]. The lipid molecule has a small polar atomic group, termed the head, and long hydrophobic aliphatic hydrocarbon chains, termed tails. Each lipid is retained in the bilayer structure due to the formation of non-covalent bonds between the head groups with adjacent lipids and near-surface water molecules, as well as hydrophobic interactions in the tail region [43].

The destruction of the cell membrane by graphene nanosheets (GN) at the molecular level was investigated by Tu et al. [40] in molecular dynamics simulations and experiments with Escherichia coli DH5a strain. They demonstrated that pristine graphene and graphene oxide nanosheets (GON) (C_20_O_2_(OH)_2_COOH) interact with 1-palmytoil-2-oleoyl-*sn*-glycero-3-phosphatidyl-ethanolamine (POPE) and mixed POPE/POPG (POPE/1-palmytoil-2-oleoyl-*sn*-glycero-3-phosphatidyl-glycerol) membranes in such that the nanosheet penetrates into the membrane bilayer, causing lipid extraction and thereby leading to bacterial membrane disruption [40]. The extraction of lipids from the 1-palmytoil-2-oleoyl-*sn*-glycero-3-phosphatidyl-choline/1-palmitoyl-2-oleoyl-*sn*-glycero-3-phospho-*L*-serine (POPC/POPS) bilayer with GN insertion was also observed in the computer simulations by Jo et al. [44]. Nanomaterials with high aspect ratios, such as asbestos fibers, nanotubes, and nanowires, may exhibit length-dependent cytotoxicity because their complete cellular uptake is impossible due to their large size [33,45,46]. The same can also be true for the case of 2D nanomaterials (2dNM), namely, the injury to the plasma membrane through the insertion of extended 2dNM into the bilayer, the complete absorption of which is impossible by geometrical factors, can also be one of the mechanisms of cellular toxicity.

To address the need in advanced quantitative in silico cell toxicity prediction models, we introduce a computational indicator of 2D nanomaterials cytotoxicity based on the analysis of “cell membrane–nanomaterial” interaction energies. We applied the suggested nanotoxicity assessment method to five different 2D nanomaterials and compared with their known data. The obtained results provide evidence that the proposed numerical indicator of cytotoxicity and the respective computational procedure could be utilized as a predictive model for safer nanomaterial design.

## 2. Method and Models 

### 2.1. Computational Indicator of Nanotoxicity of Two-Dimensional Nanomaterials (CIN_2D_)

The ability of a two-dimensional nanomaterial to disrupt the cell membrane can be determined by the way it interacts with the structural elements of the membrane. To have an effect on cell viability, minor penetration of the nanosheet into the lipid bilayer or adsorption on the membrane surface might not be sufficient, whereas severe damage to the membrane structure accompanied by significant deformation, perforation, and extraction of lipids from the bilayer, leading to loss of membrane integrity, should be considered.

The proposed approach is based on three energy parameters, which can be evaluated numerically, and the relationships between them allow us to estimate how the 2dNM interacts with the cell membrane:

(1a) $$g_{h}=\underset{\xi }{\min}\Delta G_{h}\left(\xi \right)$$

(1b) $$g_{t}=\underset{\xi }{\min}\Delta G_{t}\left(\xi \right),$$

(1c) $$g_{0}=\underset{N}{\min}\left(\frac{1}{N}g_{N}\right)$$

where $g_{h}$, $g_{t}$ are the interaction free energies of the 2dNM surface with the lipid head group and tails, respectively; $g_{0}$ is the free energy barrier of the lipid(s) extraction from the bilayer into the water solution; ξ is the reaction coordinate, which is the distance between the nanosheet plane and the interacting group of the lipid (head or tail); and $\Delta G_{h}\left(\xi \right)$, $\Delta G_{t}\left(\xi \right)$ are the Gibbs free energy changes in the interaction of the lipid head and tail parts, respectively, with the surface of the 2D nanomaterial. The aforementioned defined $g_{h}$ and $g_{t}$ are non-positive because of the application of the min(·) function and $\Delta G\left(\xi =\xi_{initial}\right)=0$ by the definition of the change. Quantity $g_{N}$, used in Equation (1c), determines the Gibbs free energy change in the hydrated bilayer when a group of *N* concatenated lipids is pulled out from it to a distance Δξ along the normal to the membrane from their equilibrium position:

(2) $$g_{N}=\underset{\Delta \xi }{\max}\Delta G_{\mathrm{ext}}^{N}\left(\Delta \xi \right)$$

**Figure 1:**
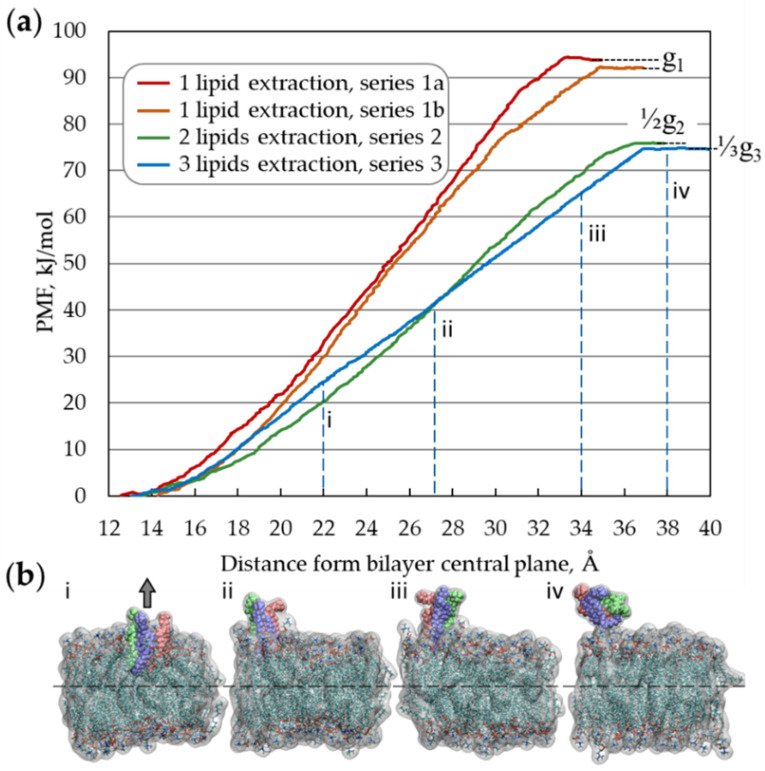
Upper estimate of the *g*_0_ obtained by calculating the potential of mean force during the extraction of a single lipid (series 1a, 1b) and a group of two (series 2) and three (series 3) adjacent lipids: (**a**) Obtained PMF profiles for series 1–3, the right-hand end points of the curves correspond to the values of $\frac{1}{N}g_{N}$ (*N* = 1, …, 3). (**b**) Four characteristic configurations of a periodic fragment of lipid membrane during the extraction of a group of three lipids (simulation series 3). Different lipids in the extracted group are represented by green, purple, and pink colors; atoms of lipids remaining in the bilayer: C—cyan, O—red, N—blue, P—brown, and H—white, water is not shown.

Thus, the value $g_{0}$ characterizes the membrane resistance to the collective extraction of lipids from it and is a constant parameter for each specific membrane (under fixed thermodynamic conditions). Notably, because the membrane is stable, $g_{N}$ >0, quantity $g_{0}$ is positive as well. If *N* = 1, the argument of the function max(·) is equal in absolute value to the single lipid embedding energy. During the collective extraction of adjacent lipids from the membrane, the specific energy (per one lipid) can be lower than that in the case of single lipid extraction because the hydrophobic contact area between a group of lipids and the remaining part of the bilayer is smaller than the contact area of one lipid multiplied by the number of lipids in the extracted group. Due to its geometry, 2dNM can simultaneously interact with many lipids and, depending on its physicochemical properties, can extract groups of concatenated lipids, which can be energetically more favorable than one-by-one extraction. In this regard, in Equation (1c), the minimal value of specific extraction energy among different sizes *N* of lipid groups is taken into consideration. The values of $g_{N}$ and $g_{0}$ are constant for each certain membrane and determined by the chemical composition of the cell membrane: its elastic properties; surface tension; temperature; and by the parameters of the cell microenvironment, such as the ionic composition and pH. These values can be predetermined and tabulated for the membranes of typical cells and bacterial membranes under standard physicochemical conditions, which can be routinely carried out and is beyond the scope of this study. However, for the particular case of a homogeneous POPC membrane under human body conditions (*T* = 310 K, *p* = 1 atm, [NaCl] = 150 mM), we obtained numerical estimates of $g_{N}$, with *N* = 1, 2 and 3, using steered molecular dynamics (SMD) simulation with potential of mean force (PMF) analysis [47,48] (Figure 1, and Supplementary Information).

The estimated values for $g_{1}$, $\frac{1}{2}g_{2}$ and $\frac{1}{3}g_{3}$ are 93.3 ± 1.5, 76.0 and 74.8 kJ/mol, respectively. The difference between $\frac{1}{2}g_{2}$ and $\frac{1}{3}g_{3}$ is less than 2%; therefore, as an estimate of $g_{0}$ for the POPC membrane, $g_{0}$ ≈ $\frac{1}{3}g_{3}$ ≈ 75 kJ/mol can be utilized further.

In terms of energy, the extraction of lipids from the bilayer onto the surface of a nanosheet, which is interacting with the cell membrane. There are two requirements for a lipid to be extracted and adsorbed with the tail group on the surface of the nanomaterial (we do not consider the adsorption by nanomaterial with a head group because such interaction leads to the adsorption of nanomaterial by the membrane surface). First, the interaction with the tails must be energetically more favorable than binding to the head group. Second, the difference in these energies must exceed the absolute value of the potential well depth $g_{0}$ of the lipid embedded in the bilayer. Additionally, what is important is that the nanosheet size is significantly larger than the bilayer thickness; otherwise, the nanomaterial will be absorbed by the hydrophobic core of the bilayer, as shown, for example, by Titov et al. [49].

There are four characteristic relationships between the above-introduced energy quantities $g_{h}$, $g_{t}$, and $g_{0}$, which determine the behavior of a 2dNM in its interaction with the cell membrane (Table 1): the case 1° $g_{h}<g_{t}$—interaction of a 2dNM with the head group is energetically more favorable than with the tails group; the case 2° $g_{h}>g_{t}$ and $g_{h}-g_{t}<g_{0}$—interaction of 2dNM with lipid tails is more favorable than with the lipid head, but the difference does not exceed the free energy barrier of lipid(s) extraction; the case 3° $g_{h}-g_{t}>g_{0}$—interaction of a 2dNM with lipid tails is more favorable than that with heads, and the difference is higher than the free energy of lipid(s) extraction from the bilayer; and in case 4°, both quantities, $\left|g_{h}\right|\;\mathrm{and}\;\left|g_{t}\right|$, are comparable or less than the level of thermal energy $k_{B}T$ (where $k_{B}$ is the Boltzmann constant, T is temperature).

As can be observed from inequalities 1°–3° in Table 1, it is convenient to describe the nanosheet behavior in its interaction with the membrane by using the following energy difference as an indicator (denoted by CIN_2D_, the computational indicator of nanotoxicity for 2dNMs):

(3) $${\mathrm{CIN}}_{2D}\;\equiv \;g_{h}-g_{t}$$

If CIN_2D_ < 0, the 2dNM interaction with the head group is energetically more favorable than with the lipid tails, and in this case, the optimal configuration is the 2dNM adsorbed on the membrane surface. If 0 < CIN_2D_ < $g_{0}$, the interaction with the hydrophobic membrane core is energetically more favorable, forcing the nanomaterial to penetrate and integrate into the lipid bilayer. With CIN_2D_ > $g_{0}$, the 2dNM tends to be inserted into the bilayer and extract lipids onto its surface, disrupting the plasma membrane structure and resulting in cytotoxicity.

### 2.2. Numerical Procedure for Rapid Assessment of CIN_2D_

**Figure 2:**
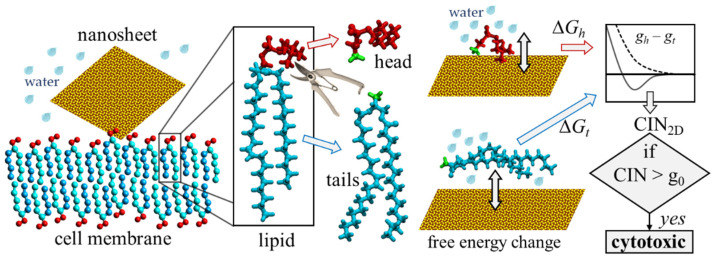
The principal idea of the computational indicator of nanotoxicity (CIN_2D_) for two-dimensional nanomaterials. The energy of interaction with the surface of the studied nanomaterial (yellow) is estimated separately for the head (red) and tail (blue) parts of the lipid. The quantitative indicator CIN_2D_ is calculated according to its definition formula. The CIN_2D_ value is then compared with the value of $g_{0}$: if CIN_2D_ exceeds $g_{0}$, the nanomaterial would impair the structural and functional integrity of the cell membrane during interaction and, thus, is cytotoxic; if CIN_2D_ below $g_{0}$, the potential toxicity cannot be predicted based on this model, and other independent studies need to be performed to confirm its safety.

The quantities $g_{h}$ and $g_{t}$ defined by Equations (1a,b) approximately correspond to the free energy adsorption by the studied 2D nanomaterial(s) in the aqueous environment of the two model molecules, which are designed on the basis of the lipid head and tail moieties, respectively. These parameters can be estimated using a simple computational procedure based on SMD simulation with PMF analysis, based on the results of two independent calculations (Figure 2): I, estimation of the free energy of adsorption of the lipid head from the water bulk solution onto the surface of the 2dNM, and II, estimation of the free energy of adsorption of the lipid tail under the same physical conditions. Next, using Equation (3), an estimate for the CIN_2D_ can be evaluated and compared with the zero value and with the value of $g_{0}$ energy.

The proposed approach has two notable methodological assumptions. First, the approach does not consider the interaction of lipids with the nanosheet edges, which may contain defects and exhibit different behavior than the regular planar surface [50,51]. Second, the difference in the accessibility and mobility of the considered lipid parts within the membrane is not considered [52]. In particular, when the lipid is embedded into the membrane, the tail part is much more constrained and screened than the head group. During the adsorption of the model molecule based on a single lipid head moiety, its phosphate and choline groups have comparable mobility, while within the membrane the phosphate group is much less mobile and therefore less able to adjust its configuration for a more energetically favorable interaction with the adsorbent surface than the choline group can. Nevertheless, we expect that such an approach in which the lipid head and tail moieties are modeled separately will provide acceptable accuracy of the presented method.

### 2.3. Graphene and Graphene Oxide Models

The pristine GN model was built with a 1.418 Å C-C bond length and potentials in Tu et al. [40]. Carbon atoms were described as Lennard–Jones particles having no electric charge. The graphene oxide model, with the chemical formula C_16_O_2_(OH)_2_COOH, was generated with our C/C++ functions library and Avogadro 1.1 software [53] in accordance with the Lerf–Klinowski model [54], so this nanosheet was characterized by an epoxy oxygen to carbon ratio of 1:8 and a hydroxyl and carboxyl groups to carbon ration of 1:8 and 1:16, respectively. Force field parameters were from Tang et al. [55].

### 2.4. Layered Double Hydroxide and Aloohene Models

The Mg/Al-LDH nanosheet model with the chemical composition Mg_2_Al(OH)_6_^+^ × Cl^−^ × *n*H_2_O was constructed using data from Krivovichev et al. [56]. The geometry of the aloohene flat domain was built with the unit cell structure in Noel et al. [57], as we have carried out in our earlier studies [6,58]. Aloohene and Mg/Al-LDH model parameterization was performed in accordance with the CLAYFF force field [59], but the null Lennard–Jones parameters for hydroxyl hydrogen were replaced by *r*_0_ = 0.449 Å, *ε* = 0.046 kcal/mol from the CHARMM force field [60], in analogy with paper [61], aiming for proper interactions between the CLAYFF and CHARMM subsystems of the model.

### 2.5. Boron Nitride Nanosheet Models

Hexagonal boron nitride nanosheet (BNN) models were constructed with a 1.446 Å B-N bond length; the Lennard–Jones parameters were chosen to be equal to the corresponding values of other studies [62,63], which were used for hexagonal boron nitride nanomaterials. The partial atomic charges of B and N atoms of boron nitride compounds are strong environmentally dependent [64]. Therefore, we tested two sets of partial atomic charges for B and N atoms—PAC-I Q_B_ = 1.05 e, Q_N_ = −1.05 e; PAC-II Q_B_ = 0.5 e, Q_N_ = −0.5 e—in accordance with Ref. [65]. For instance, two PAC sets of ±1.05 e and ±0.4 e were also used for the boron nitride 2dNM model [66].

### 2.6. Lipid and Water Models

The 1-palmytoil-2-oleoyl-*sn*-glycero-3-phosphatidyl-choline phospholipid was considered as a representative building unit of the cell membrane. The head and tail parts of the lipid molecule were modeled in accordance with the CHARMM36 force field [67]. An additional methyl group (green atoms in Figure 2), used to terminate the molecule instead of the absent part, was parameterized as a CTL3-type carbon atom with a partial charge of −0.27 e, with three HAL3-type hydrogen atoms with a charge of +0.09 e.

Water was modeled using the modified TIP3P model [68], which is compatible with the CHARMM force field. 

### 2.7. Simulation Details

The simulation boxes dimensions were as follows: 32 × 38 × 45 Å (for GN), 30 × 34 × 56 Å (GON), 32 × 37 × 44 Å (Mg/Al-LDH), 30 × 37 × 33 Å (aloohene), and 33 × 39 × 43 Å (BNN). Periodic boundary conditions were applied in all directions. The total number of atoms was approximately 3700–5500, depending on the simulation case. The heavy (non-hydrogen) atoms of all nanosheets, except the carboxyl and hydroxyl groups of GON, were motionless during the simulation time. Simulations were performed for the system under the *Np_z_T* conditions at human body temperature (310 K) and 1 atm pressure. Full electrostatics were treated using the particle–particle–particle–mesh (PPPM) algorithm [69,70]. The SHAKE algorithm [71] for all bonds, in which the hydrogen atom is involved, was used to increase the integration time step up to 2 fs. For estimating the free energy change, bidirectional constant velocity SMD simulations of adsorption–desorption were conducted. The bidirectional SMD results in a faster convergence than a unidirectional one [72,73]. Furthermore, we used a slow velocity constant of 0.0001 Å/ps, which allows adsorbate molecules to adopt various configurations during steered adsorption to find energetically favorable variants. The stiffness of the virtual spring was set to 1000 kJ/(mol·Å^2^). Each estimate of a ΔG(ξ) profile for GN, Mg/Al-LDH, aloohene, and a BNN was obtained using four PMF(ξ) profiles (two forced adsorption curves and two forced desorption curves). To obtain ΔG(ξ) for the GON case, we calculated eight PMF profiles—two forced adsorption curves and two forced desorption curves obtained for each of the nanosheet surfaces—which have different surface structures with randomly placed oxygen-containing groups (i.e., hydroxyl, carboxyl, and epoxy groups). To make the procedure of cytotoxicity assessment more rapid and to take into the account both the use of bidirectional protocol and the low velocity constant, we utilized only four or eight PMF profiles for each pair of adsorbate–adsorbent to estimate the free energy change. A more accurate estimation may be also obtained using several tens of trajectories and Jarzynski equation [74], or other techniques of thermodynamic integration [47,75].

Simulations were conducted using the resources of two supercomputers: “Lomonosov-2″ (Lomonosov Moscow State University, Russia) [76,77] and “Zhores” of the Center for Computational and Data-Intensive Science and Engineering, Skolkovo Institute of Science and Technology (CDISE, Skoltech, Russia) [78]. The parallel multifunctional simulation package LAMMPS (Sandia National Laboratory, USA) [79,80] was used for all simulations. Molecular graphics were prepared using the Visual Molecular Dynamics 1.9.2 package [81].

## 3. Results and Discussion

A suggested rapid assessment procedure for the introduced indicator CIN_2D_ was applied to the five most famous 2dNMs, which are important for biomedical applications: graphene nanosheets (GN) and graphene oxide nanosheets (GON) [82] and references within, BNN [83,84], the flat domain of the aloohene [6,85,86,87], and a single layer of [Mg_2_Al(OH)_6_ × Cl] layered double hydroxide (Mg/Al-LDH) [88,89]. One of the most commonly used electrically neutral phospholipids, POPC lipid, was used as the test one.

The chemical structure of the POPC lipid is N(CH_3_)_3_–CH_2_–CH_2_–PO_4_–C* H_2_–<tails>, where <tails> means palmytoil–CH_2_–C** H–oleoyl, and C* is bonded with C**. For structural reasons, the lipid was divided for the numerical experiment as follows: the head part is phosphatidylcholine with the phosphate group terminated by a neutral methyl group N(CH_3_)_3_–CH_2_–CH_2_–PO_4_–CH_2_–CH_3_ instead of long aliphatic chains, and the tail part is CH_3_–<tails>, with an uncharged methyl group (green atoms in Figure 2) added instead of the polar head.

**Figure 3:**
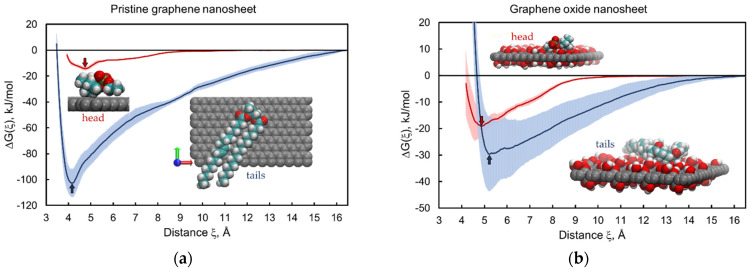
Free energy change profiles for adsorption–desorption of the lipid head (red) and tail (blue) parts on graphene (**a**) and graphene oxide (**b**) nanosheets. Inserts correspond to molecular configurations in the adsorbed state in local free energy minima (red and blue arrows for head and tail parts, respectively). A standard deviation corridor is depicted around each profile by half-transparent filling of the same color. Color code: graphene carbon (gray), lipid carbon (cyan), oxygen (red), hydrogen (white), nitrogen (blue), and phosphorus (mustard). Water is not shown.

**Figure 4:**
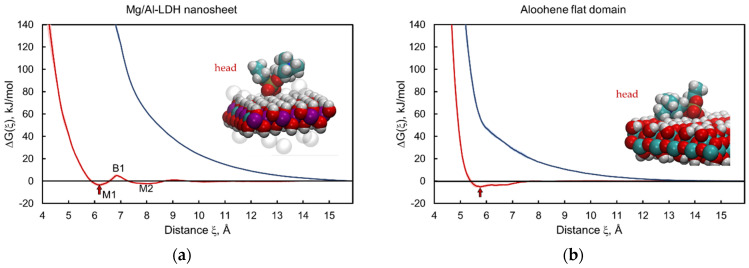
Free energy change profiles for adsorption–desorption of the head (red) and tail (blue) parts of the lipid on the Mg/Al-LDH nanosheet (**a**) and aloohene flat domain (**b**). Inserts correspond to the adsorbed states of the head part at the ∆*G_h_* local minima (red arrows): M1 for Mg/Al-LDH (configuration in the M2 state is in Supplementary Figure S1) and M for aloohene (see Supplementary Table S1). Adsorption of lipid tails was not observed and the free energy infinitely increases (blue curves) when the lipid tail part gets closer to the hydroxides surfaces. Color code: magnesium (purple), aluminium (cyan), chlorine ions (transparent); other colors are the same as in Figure 3.

**Figure 5:**
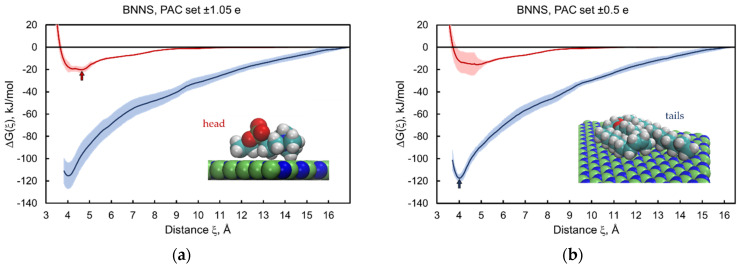
Free energy change profiles for adsorption–desorption of the head (red) and tail (blue) parts of the POPC lipid on boron nitride nanosheets. Results obtained for two sets of partial atomic charges: PAC-I ± 1.05 e (**a**) and PAC-II ± 0.5 e (**b**). Color code: boron (green), nitrogen (blue); other colors are the same as in Figure 3. Alternative views of the lipid tail part in the adsorbed state are depicted in Supplementary Figure S2.

We performed bidirectional constant velocity SMD simulations of adsorption–desorption to obtain the estimates of adsorption free energies separately for the head and tail parts of the lipid onto the nanomaterial surface. The calculated profiles of the free energy change $\Delta G_{h}\left(\xi \right)$ and $\Delta G_{t}\left(\xi \right)$ as a function of the distance between the center of mass of the adsorbate molecule and the central plane of the nanosheets are shown in Figure 3, Figure 4 and Figure 5 for all five 2dNM chosen. The red curves correspond to the profiles of the free energy change for the adsorption–desorption of the lipid head, and the blue curves are the same for the tails part of the lipid. A local minimum near the nanomaterial surface on the free energy change profile is interpreted as the adsorbed state of the molecule, and the free energy change value at this point is an estimate of the free energy of adsorption that determines the energy of interaction of the lipid head $g_{h}$ or tail $g_{t}$ with the surface of the nanomaterial. The configurations of the head and tail parts of the lipid in the adsorbed state on the surface of the 2dNM are shown in the inserts in each figure (quantitative estimates are summarized in Table 2).

### 3.1. Graphene and Graphene Oxide Nanosheets

The results of the estimations of the ∆*G_h_* and ∆*G_t_* profiles for the head and tail moieties of the POPC lipid interacting with a pristine GN are presented in Figure 3a. The ∆*G_t_* profile (blue curve) is below ∆*G_h_* (red curve), which indicates that the interaction of the hydrophobic GN with the hydrophobic tail part is energetically more favorable than that with the hydrophilic head part. However, notably, ∆*G_h_* also has a local minimum (Figure 3a, red curve), which corresponds to the interaction of hydrophobic methyl groups of the head with the nanosheet surface (see the inset in Figure 3a).

The CIN_2D_ estimate obtained from these free energy profiles is 88 ± 13 kJ/mol, which is larger than $g_{0}$, indicating the cytotoxicity of the given nanomaterial (see Table 1). Indeed, according to the free energy estimates ∆*G_h_* and ∆*G_t_*, the GNS, while interacting with the membrane, can adsorb the head group of the POPC lipid and then displace the lipid out of the bilayer onto the nanosheet surface, forming energetically more favorable bonds with hydrophobic tails of the lipid. Depending on the size, the nanosheet can also be inserted into the bilayer with subsequent extraction of lipids onto its surface, because CIN_2D_ > $g_{0}$, and particularly, |$g_{t}$| > $g_{0}$. This effect of the nanosheet on the membrane determines its cytotoxicity, which agrees with published results for graphene [39].

Notably, because the values $g_{h}$ and $g_{t}$ are non-positive, the condition CIN_2D_ > $g_{0}$ is stronger than |$g_{t}$| > $g_{0}$, because the second inequality is a consequence of the first inequality.

$${\mathrm{CIN}}_{2D}>g_{0}\;⟺-g_{t}>g_{0}-g_{h}\;\Rightarrow \;\left|g_{t}\right|>g_{0}+\left|g_{h}\right|\;\Rightarrow \;\left|g_{t}\right|>g_{0}$$

In contrast to pure graphene, graphene oxide has a non-regular surface structure with a variety of randomly placed oxide forms on its surface. Here, we consider a graphene oxide nanosheet (GON) with the chemical formula C_16_O_2_(OH)_2_COOH. The presence of hydrophilic polar groups on the GON’s surface affected the energy of interaction with both the lipid head and tail moieties. Most notably, ∆*G_h_* (Figure 3b, red curve) has a deeper minimum than in the case of pristine graphene, −19 ± 4 kJ/mol versus −14 ± 2 kJ/mol, which indirectly indicates a *higher hydrophilicity* of the GON. The ∆*G_t_* profile (Figure 3b, blue curve) lies well above the same curve for pristine graphene (Figure 3a), with a difference of 73 ± 25 kJ/mol, indicating a *lower hydrophobicity* of graphene oxide compared with GN.

An estimate of CIN_2D_ = 10 ± 18 kJ/mol was positive but did not exceed the value of the specific free energy of lipid extraction, CIN_2D_ < $g_{0}$. Notably, in this case, the magnitude of the error calculated by the standard deviation is greater than the CIN_2D_ estimate, due to which the CIN_2D_ value obtained for graphene oxide introduces uncertainty to the *prediction* of how the GON will interact with the cell membrane. Nevertheless, the obtained result suggests that for the considered GON fragment of composition C_16_O_2_(OH)_2_COOH, the interaction with the lipid tail part is energetically more favorable than with the head part. Thus, the GON will tend to insert into the lipid membrane, leading to an increase in the contact area with the hydrophobic core of the bilayer. There will be no substantial extraction of lipids from the membrane under such conditions because the state of the lipid within the membrane is energetically more favorable than its adsorption on the surface of the GON. In this case, the cell membrane can be mechanically damaged depending on the nanosheet’s size; therefore, the condition 0 < CIN_2D_ < $g_{0}$ does not determine toxicity but indicates an energetically favorable state in which the nanosheet is inserted into the membrane.

Liao et al., in their in vitro studies, showed that graphene oxide and GN exhibited cytotoxicity in relation to red blood cells [90]. By contrast, Chang et al. [91] reported that GON are not obviously cytotoxic to human lung carcinoma epithelial cells A549 and do not tend to enter these cells. According to the results of Sasidharan et al., graphene functionalized with carboxyl groups demonstrated good biocompatibility in contrast with pristine graphene [92]. This finding indicates that the ability of graphene oxide to destroy the cell membrane strongly depends on the degree of nanosheet oxidation: the higher the oxidation degree, the lower the capacity for membrane disruption. These findings are consistent with those of in vitro experiments on fullerenes [14], in which they found that nanoscale aggregates of pristine C_60_ fullerenes are cytotoxic to human skin (HDF) and liver carcinoma (HepG2) cell lines but that functionalized C_60_ with carboxylic groups and water-soluble fullerene derivatives Na^+^_2–3_[C_60_O_7-9_(OH)_12-15_]^(2–3)−^ are less cytotoxic to the same cells, whereas fullerene hydroxide C_60_(OH)_24_ demonstrates no cytotoxicity [14].

In summary, the condition 0 < CIN_2D_ < $g_{0}$ indicates possible size-dependent toxicity of the nanomaterial, and the CIN_2D_ value for graphene depends on the degree of its surface oxidation; and, notably, CIN_2D_ for pure graphene is significantly larger than that for graphene oxide.

### 3.2. Layered Double Hydroxide Nanosheet and Aloohene Flat Domain

In contrast with graphene and graphene oxide, Mg/Al-LDH and aloohene are hydrophilic, and their surfaces are completely formed by polar OH groups. The free energy change profile of Mg/Al-LDH interacting with the POPC lipid head has several local minima separated by potential barriers (Figure 4a, red curve). Its behavior is the most complex of all the profiles studied in this work (see Supplementary Table S1 for detailed positions and values of local free energy minima and energy barriers). The position of the local minima reflects the structure of the near-surface water layers that hydrate the surface of the hydrophilic material. As observed from the ∆*G_h_* and ∆*G_t_* profiles, the results for Mg/Al-LDH (Figure 4a) and aloohene (Figure 4b) are opposite the case of graphene, which we expected.

The ∆*G_t_* profiles (Figure 4, blue curves) for the tail part of the lipid lie above the ∆*G_h_* curves for the head (Figure 4, red curves). The presence of local minima on the free energy profile for the head part and their absence for hydrophobic tails means that neither the Mg/Al-LDH nanosheet nor aloohene tends to penetrate into the hydrophobic core of the bilayer but is adsorbed on the cell membrane surface. Notably, during such adsorption, the wrapping of nanomaterial by the membrane is possible, followed by its cellular uptake [93]. Additionally, Ladewig et al. observed the uptake of the layered double hydroxide nanoparticles into mammalian cells via endocytosis [94].

In both cases, CIN_2D_ has a negative value of −3.5 ± 1.5 kJ/mol and −5.1 ± 1.3 kJ/mol for Mg/Al-LDH and aloohene, respectively. The obtained CIN_2D_ estimates only imply that the cell membrane will not be disrupted and that its integrity will be preserved; other mechanisms of toxicity cannot be identified using the described approach. Additionally, laboratory tests on the cytotoxicity of layered double hydroxides of various metals have indicated that such hydroxides have no or very low cytotoxicity [95,96]. In vivo studies have shown that [Mg_2_Al(OH)_6_^+^ Cl^−^]-LDH and [Zn_2_Al(OH)_6_^+^ Cl^−^]-LDH exhibit biocompatibility, non-toxic, and non-immunogenic properties [97]. For aloohene, there is no experimental evidence of its destructive effect on the cell membrane. In vitro test results revealed that aloohene-like 2D structures of aluminum oxyhydroxide have low cytotoxicity against the normal cell line L929 [98]. Lerner et al. showed that aloohene inhibits cancer cell growth and potentiates chemotherapeutic drugs; however, aloohene action is associated with the dysregulation of ion balance in the cancer cell microenvironment, rather than with cell wall disruption [6].

### 3.3. Boron Nitride Nanosheets

Low-dimensional hexagonal boron nitride nanomaterials have drawn considerable attention due to their potential applications in nanomedicine [99,100,101,102]. Therefore, we considered the boron nitride nanosheet (BNN) for an assessment by using our approach.

Because of a strong dependence of the partial atomic charges of boron nitride nanomaterials on the environment [64], we examined two sets of partial atomic charges selected by using a method in Hilder and Gaston [65]: ±1.05 e (PAC-I) and ±0.5 e (PAC-II), with a negative value for nitrogen and a positive value for boron atoms. Despite the significant difference in the partial charges, the obtained estimates of the free energy of adsorption for the head and tail parts of the POPC lipid are close to each other (Figure 5a,b, blue curves). The difference in the results for the lipid tail part did not exceed 3% (Table 2, Supplementary Table S1). The profiles for the lipid head also coincide, with allowance for measurement error, for cases with different sets of partial charges. Moreover, the results for the BNN are similar to those for pristine graphene (compare Figure 3a and Figure 5a,b).

The calculated CIN_2D_ values for the BNN were 95 ± 15 kJ/mol (PAC-I) and 102 ± 10 kJ/mol (PAC-II). Both were larger than *g*_0_, as well as for the case of pristine graphene. Thus, the BNN interacting with the lipid membrane behaves similarly to the GN, i.e., it tends to insert into the bilayer, causing a substantial extraction of lipids from the membrane. This outcome is in good agreement with the experimental and simulation results recently reported [103,104]. In particular, using a combined approach with both experimental and theoretical techniques, Zhang et al. demonstrated that BNN cause membrane degradation of the Gram-negative bacteria *Escherichia coli* [103], and Xie et al. demonstrated that BNN can destroy the cell membranes of red blood cells [104].

**Figure 6:**
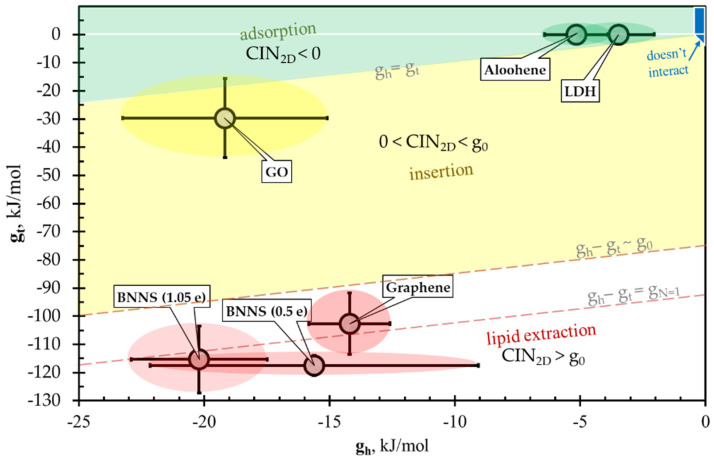
CIN_2D_ diagram $g_{t}$ versus $g_{h}$ comprises four regions: “no interaction” (blue region, schematically), “adsorption” of nanosheet by membrane (green region), “insertion” (yellow) and “lipid extraction” (white region). Ellipses for aloohene and Mg/Al-LDH nanosheets are inside the adsorption region (green ellipses). Pristine graphene and both models of BNN (red ellipses) are inside the lipid-extraction region, where CIN_2D_ is larger than $g_{0}$ that means these nanosheets are cytotoxic. Graphene oxide nanosheet (yellow ellipse), having chemical composition C_16_O_2_(OH)_2_(COOH)_1_, is inside the “insertion” region.

Additionally, what is notable is that the results of some experimental [105,106,107,108] and theoretical [63,66] studies have indicated a lower cytotoxicity and better biocompatibility of boron nitride-based nanomaterials such as boron nitride nanotubes than with similar carbon-based nanomaterials; however, in the case of nanosheets, the proposed CIN_2D_ approach reveals no difference in toxicity between pristine graphene and boron nitride; both nanomaterials can induce cell membrane disruption (Table 2 and Figure 6).

In summary, according to our results and reasoning, the behavior of the BNN with respect to the POPC lipid bilayer should be similar to that of the GN, i.e., it causes lipid extraction.

### 3.4. CIN_2D_ Diagram

The relationships among the introduced energy parameters $g_{h}$, $g_{t}$, and $g_{0}$, which can be used to predict the 2dNM behavior interacting with the cell membrane, may be conveniently represented as a $g_{h}$ versus $g_{t}$ phase diagram (Figure 6). With this representation, the phase plane is conventionally divided into four regions of characteristic nanomaterial behavior (Table 1):

1. CIN_2D_ < 0, nanomaterial adsorption on the membrane surface (Figure 6, green region).
2. 0 < CIN_2D_ < $g_{0}$, nanomaterial insertion into the bilayer (Figure 6, yellow).
3. CIN_2D_ > $g_{0}$—disruptive lipid extraction (Figure 6, white).
4. |$g_{h}$|+|$g_{t}$| < *k*_B_*T* ~ no significant interaction (Figure 6, blue).

According to the results of the performed SMD simulations, the five considered nanomaterials fell into three different groups with respect to the CIN_2D_ value. Mg/Al-LDH and aloohene are in the “green” group with the negative value of CIN_2D_; the graphene oxide nanosheet is in the “yellow” group with the above-discussed uncertainty; and the pristine GN and BNN are in the “red” group with a CIN_2D_ value exceeding the specific energy of lipid extraction, CIN_2D_ > $g_{0}\;$(Figure 6). Notably, the proposed approach, which predicts the mechanism of 2D nanomaterial cytotoxicity associated with cell membrane disruption, can be extended to predict the interaction of 2D nanomaterials with intracellular organelles, whose membranes are organized similarly to the cell membrane.

## 4. Conclusions

In summary, a numerical indicator of cytotoxicity of 2D nanomaterials CIN_2D_ was proposed, and a computational procedure for its fast assessment was designed based on the analysis of the free energy of adsorption separately for the head and tail parts of the membrane-forming lipid. The CIN_2D_ estimate obtained by this approach reflects the energy features of the nanosheet–membrane interaction. An indicator value above the critical value CIN_2D_ > $g_{0}$ indicates the potential cytotoxicity of a 2D nanomaterial; CIN_2D_ < $g_{0}$ suggests that the nanomaterial interacting with the cell wall is less likely to induce disruption of the lipid bilayer, although the nanomaterial may be toxic because of a different mechanism, independent of the cell membrane damage.

When CIN_2D_ is lower than $g_{0}$, the proposed numerical indicator shows which mutual configuration between the 2D nanomaterial and the membrane is energetically more favorable. If CIN_2D_ > 0, the contact area between the nanomaterial and the hydrophobic region of the bilayer tends to increase; in this case, the most energetically favorable configuration is the insertion into the bilayer with a transmembrane orientation or penetration into the bilayer interior with a parallel membrane orientation that can be determined by the nanomaterial size and shape. If CIN_2D_ < 0, the nanomaterial is more likely to be adsorbed on the membrane surface; this does not exclude the cellular uptake of the nanomaterial, which depends on many factors, including the nanoparticle size and structure of the edge zones.

The presented approach was applied to five 2D nanomaterials (pure graphene, graphene oxide, Mg/Al-LDH, aloohene, and hexagonal boron nitride), which are promising for biomedical applications. Independent published data on the interaction of these nanomaterials with cell membrane were used to verify the developed CIN_2D_ indicator approach. We showed that the predictions obtained in our study using the described numerical procedure are in good agreement with the published experimental data on the cytotoxicity of nanosheets. Therefore, this approach can be considered a useful tool for computational nanotoxicology and thus assists in the development of engineered nanomaterials with controlled or/and safe bioactivity.

We hope that the proposed CIN_2D_ indicator and the method of its fast assessment will contribute to the assortment of tools for the computer-aided design of new nanomaterials and for the in silico modeling approach in general.

## Figures and Tables

**Table 1:** Relationships between $g_{t}$, $g_{h}$ and $g_{0}$, characterizing the behavior of a 2D nanomaterial in its interaction with the lipid membrane (the figures in the right column schematically show possible mutual configurations: water—light-blue, lipid head—red, lipid tail—purple, 2dNM—black contour, selected lipid—green).

	Relationship	Description	Behavior
1°	$g_{h}-g_{t}$ < 0	Interaction with the head group is energetically more favorable than with the tails, leading to nanosheet adsorption by the membrane surface	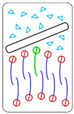 *Adsorption* of nanomaterial on membrane surface
2°	$0<g_{h}-g_{t}<g_{0}$	Nanosheet insertion into the bilayer is energetically favorable. Size-dependent mechanical disruption of membrane is also possible	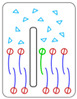 *Insertion* of nanomaterial into bilayer
3°	$g_{0}$ < $g_{h}-g_{t}$	Nanosheet insertion into the bilayer with lipid extraction and membrane disruption	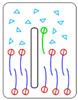 Disruptive *lipid extraction*
4°	$\left|g_{h}\right|,\;\left|g_{t}\right|≲k_{B}T$	Nanosheet does not, or weakly, interacts with the cell membrane—non-critical impact	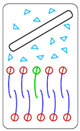 *No interaction*

**Table 2:** Computational indicator of nanotoxicity CIN_2D_ for different 2D nanomaterials.

Nanomaterial	*g_h_*, kJ/mol	*g_t_*, kJ/mol	CIN_2D_, kJ/mol	Relationship	Prediction
GN	−14 ± 2	−103 ± 11	88 ± 13	$\mathrm{CIN}\;>\;g_{0}$	lipid extraction
GON	−19 ± 4	−30 ± 14	10 ± 18 *	$0\;<\;\mathrm{CIN}\;<\;g_{0}$	insertion into bilayer
Mg/Al-LDH	−3.5 ± 1.4	0.00 ± 0.12	−3.5 ± 1.5	CIN < 0	adsorption by bilayer
Aloohene	−5.2 ± 1.3	−0.03 ± 0.05	−5.1 ± 1.3	CIN < 0	adsorption by bilayer
BNN (PAC ± 1.05 e)	−20 ± 3	−115 ± 12	95 ± 15	$\mathrm{CIN}\;>\;g_{0}$	lipid extraction
BNN (PAC ± 0.5 e)	−16 ± 7	−118 ± 4	102 ± 10	$\mathrm{CIN}\;>\;g_{0}$	lipid extraction

* Magnitude of the error estimated by standard deviation is larger than the CIN_2D_ estimate, that is why obtained for graphene oxide CIN_2D_ cannot provide strong confidence in the cytotoxicity of modelled GON.

## Data Availability

All necessary data and models will be uploaded to GitHub repository https://github.com/AATsukanov/CIN2D (11 February 2022).

## Supplementary Materials

The following supporting information can be downloaded at: https://www.mdpi.com/article/10.3390/nano12040650/s1. Simulation details, Figure S1: Configurations of head part of POPC lipid adsorbed at local minima on the surface of Mg/Al-LDH, Figure S2: Typical configurations of tails part of POPC lipid adsorbed on the surface of BNN, Table S1: Quantitative parameters of ∆G profiles estimated for head and tail moieties of the POPC lipid interacting with the two-dimensional nanomaterials. Refs. [47,67,69,70,109,110,111,112,113] are cited in Supplementary Materials.
