# Supplementary material for: Computational Indicator Approach for Assessment of Nanotoxicity of Two-Dimensional Nanomaterials
Source: Nanomaterials (Basel). 2022 Feb 15;12(4):650. doi: 10.3390/nano12040650 (PMC8879952; doi:10.3390/nano12040650)
Supplement: Supplementary file 1 [file nanomaterials-12-00650-s001.zip › nanomaterials-1539659-SI-proof-final.pdf]

nanomaterials

Supplementary Materials

Computational Indicator Approach for Assessment of Nanotoxicity of Two-Dimensional Nanomaterials

Alexey A. Tsukanov [1,2,*], Boris Turk [3], Olga Vasiljeva [3,*,†] and Sergey G. Psakhie [2,‡]

1 Center for Computational and Data-Intensive Science and Engineering (CDISE), Skolkovo Institute of Science and Technology (Skoltech), 121205 Moscow, Russia
2 Institute of Strength Physics and Materials Science of SB RAS, 634055 Tomsk, Russia;
3 Department of Biochemistry and Molecular and Structural Biology, Jozef Stefan Institute, SI-1000 Ljubljana, Slovenia; boris.turk@ijs.si
* Correspondence: a.tsukanov@skoltech.ru (A.A.T.); olga.vasiljeva@ijs.si (O.V.)
† Present Address: CytomX Therapeutics, Inc., South San Francisco, CA 94080, USA.
‡ Deceased.

1. SIMULATION DETAILS

Calculation of the free energy of lipid extraction

Constant velocity steered molecular dynamic (SMD) [1] simulations were performed to estimate free energy change during the single and a group of 2 and 3 lipids complete extraction from POPC bilayer into the bulk water solution.

Initial dimensions of the simulation box were 60 × 60 × 154 Å. The model membrane was composed of 106 lipids (53 in each leaflet). The membrane was oriented in XY plane and its center of mass was restrained to the plane Z = 0 by the stiff spring with a constant 30000 kJ/(mol·Å^2). Membrane model was generated using CHARMM-GUI v2.0 (http://www.charmm-gui.org) [2] and was parameterized with CHARMM36 force field [3]. Total number of atoms was 52 396, including 12 708 water molecules and 34 Na^+ and 34 Cl^- ions. The periodic boundary conditions were set along all the directions. System was modelled as isothermal-isobaric (NpT) ensemble at T = 310 K and p = 1 atm with a Nose–Hoover algorithm [4,5]. Dimensions of simulation box were changeable to provide target pressure. X and Y dimensions were coupled, while Z was changing independently. Long-range electrostatics was computed with particle-particle particle-mesh (PPPM) algorithm [6,7].

In order to estimate free energy change (ΔG) of the system during the N lipids extraction, the PMF was calculated as the sum of the works done by N virtual springs with constant of 2000 kJ/(mol·Å^2), with one end of which attached to the center of mass of one from N lipids in the group and the other end moved at a constant velocity 0.1 Å/ns away from membrane and along Z. The X and Y components of the virtual force were zero.

The molecular dynamics models and other simulations-related data can be found at [8,9].

2. SUPPLEMENTAL RESULT DETAILS

Table S1. Quantitative parameters of **ΔG** profiles estimated for head and tail moieties of the POPC lipid interacting with the two-dimensional nanomaterials. M – point of local minimum, **B** – energy barrier, **ΔG** – an estimation of the free energy change, SD – standard deviation of **ΔG** (error estimation), ξ – distance between nanosheet and adsorbate molecule center of mass.

Nanomaterial	Lipid part	Min./Barrier	ΔG, kJ/mol	SD, kJ/mol	ξ, Å
**GN**	head	M	-14.2	1.62	4.75
	tails	M	-102.69	10.92	4.13
**GON**	head	M	-19.17	4.08	4.84
	tails	M	-29.64	14.04	5.21
**Mg/Al-LDH**	head	M4	-0.3	0.19	12.64
		M3	-0.61	0.28	10.17
		**B**2	1.1	0.37	9.06
		M2	-2.29	0.61	8.08
		**B**1	5.09	1.08	6.84
		M1	-3.47	1.41	6.17
	tails	(M)	0	0.12	(inf)
**Aloohene**	head	M'	-3.78	1.23	6.32
		M	-5.16	1.26	5.76
	tails	(M)	-0.029	0.046	(16.04)
**BNN (±0.5 e)**	head	M	-15.61	6.55	4.82
	tails	M	-117.51	3.63	4.02
**BNN (±1.05 e)**	head	M	-20.2	2.71	4.66
	tails	M	-115.35	11.91	4.02

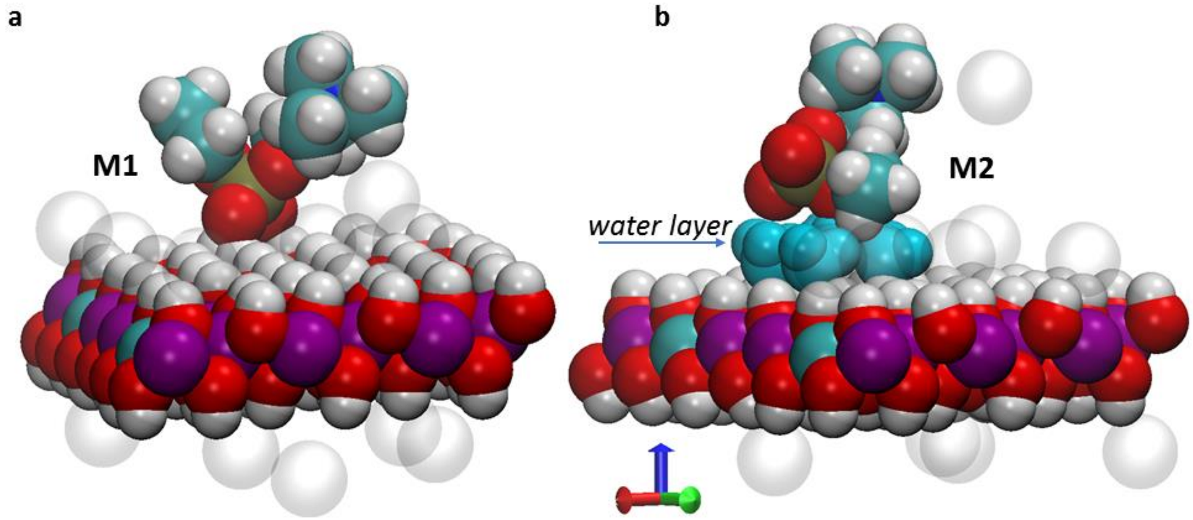

Figure S1. Configurations of head part of POPC lipid adsorbed at local minima M1 (**a**) ξ = 6.17 Å and M2 (**b**) ξ = 8.08 Å on the surface of Mg/Al-LDH. Water is not shown, except several molecules, which form single-molecule-thin water layer between LDH surface and PO_4-group of a lipid head. Colors: aluminum – cyan, magnesium – purple, carbon – cyan, oxygen – red, hydrogen – white, phosphorus – golden, nitrogen – blue, chlorine ions – transparent, water – light blue.

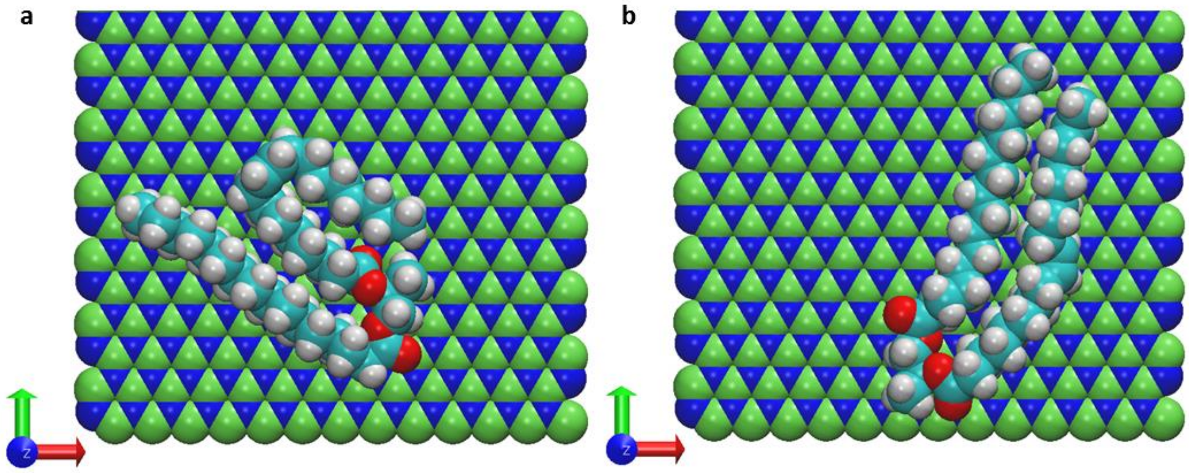

Figure S2. Typical configurations of tails part of POPC lipid adsorbed on the surface of BNN (near point of local minimum M, ξ = 4.02 Å). Colors: boron – green, nitrogen – blue, carbon – cyan, oxygen – red, hydrogen – white.

References

1. Izrailev, S.; Stepaniants, S.; Isralewitz, B.; Kosztin, D.; Lu, H.; Molnar, F.; Wriggers, W.; Schulten, K. *Steered Molecular Dynamics. Computational Molecular Dynamics: Challenges, Methods, Ideas*; Springer: Berlin, Germany, 1997; pp. 39–65.
2. Jo, S.; Lim, J.B.; Klauda, J.B.; Im, W. CHARMM-GUI Membrane Builder for mixed bilayers and its application to yeast membranes. *Biophys. J.* **2009**, *97*, 50–58.
3. Klauda, J.B.; Venable, R.M.; Freites, J.A.; O'Connor, J.W.; Tobias, D.J.; Mondragon-Ramirez, C.; Vorobyov, I.; MacKerell, A.D., Jr.; Pastor, R.W. Update of the CHARMM all-atom additive force field for lipids: Validation on six lipid types. *J. Phys. Chem. B* **2010**, *114*, 7830–7843.
4. Nosé, S. A molecular dynamics method for simulations in the canonical ensemble. *Mol.Phys.* **1984**, *52*, 255–268.
5. Hoover, W.G. Canonical dynamics: Equilibrium phase-space distributions. *Phys. Rev.* A **1985**, *31*, 1695–1697.
6. Hockney, R.W.; Goel, S.P.; Eastwood, J.W. A 10000 particle molecular dynamics model with long range forces. *Chem. Phys. Lett.* **1973**, *21*, 589–591.
7. Hockney, R.W.; Eastwood, J.W. *Computer Simulation Using Particles*; CRC Press, Boca Raton, USA: 1988.
8. Molecular Dynamics Models and Related Data Will Be Available at GitHub Repository via Link. Available online: https://github.com/AATsukanov/CIN2D (accessed on 11 February 2022).
9. Available online: https://tsukanov-lab.moy.su (accessed on 11 February 2022).
